# Tetra­aqua­{1-[(1*H*-1,2,3-benzotriazol-1-yl)meth­yl]-1*H*-1,2,4-triazole}sulfato­cadmium dihydrate

**DOI:** 10.1107/S1600536811032442

**Published:** 2011-08-17

**Authors:** Yu-xian Li, Da-wei Li, Dong Zhao

**Affiliations:** a Pharmacy College, Henan University of Traditional Chinese Medicine, Zhengzhou 450008, People’s Republic of China; b ZhengZhou Trade and Industry Schools, Zhengzhou 450000, People’s Republic of China; c Department of Chemistry, Zhengzhou University, Zhengzhou 450052, People’s Republic of China

## Abstract

In the title complex, [Cd(SO_4_)(C_9_H_8_N_6_)(H_2_O)_4_]·2H_2_O, the Cd^II^ ion is six-coordinated by one N atom from a 1-[(1*H*-1,2,3-benzotriazol-1-yl)meth­yl]-1*H*-1,2,4-triazole ligand and by five O atoms from four water mol­ecules and one monodentate sulfate anion in a distorted octa­hedral geometry. The sulfate tetra­hedron is rotationally disordered over two positions in a 0.651 (12):0.349 (12) ratio. In the crystal, adjacent mol­ecules are linked through O—H⋯O and O—H⋯N hydrogen bonds into a three-dimensional network.

## Related literature

For background to complexes based on triazolyl or benzotriazolyl ligands, see: Meng *et al.* (2009 ▶); Yang *et al.* (2011 ▶).
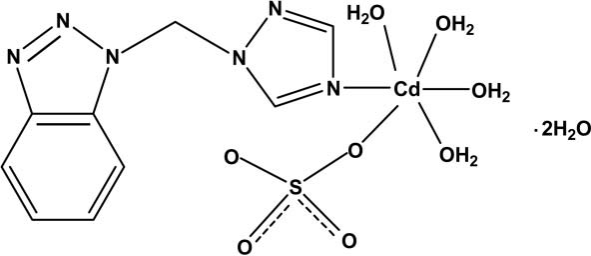


## Experimental

### 

#### Crystal data

[Cd(SO_4_)(C_9_H_8_N_6_)(H_2_O)_4_]·2H_2_O
*M* *_r_* = 516.77
Triclinic, 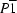

*a* = 7.7154 (15) Å
*b* = 8.0667 (16) Å
*c* = 16.369 (3) Å
α = 100.12 (3)°
β = 91.64 (3)°
γ = 112.38 (3)°
*V* = 922.3 (3) Å^3^
*Z* = 2
Mo *K*α radiation
μ = 1.36 mm^−1^
*T* = 293 K
0.19 × 0.17 × 0.14 mm

#### Data collection

Rigaku Saturn CCD diffractometer
Absorption correction: multi-scan (*REQAB*; Jacobson, 1998 ▶) *T*_min_ = 0.782, *T*_max_ = 0.832
8812 measured reflections
3608 independent reflections
3361 reflections with *I* > 2σ(*I*)
*R*_int_ = 0.020

#### Refinement

*R*[*F* ^2^ > 2σ(*F* ^2^)] = 0.025
*wR*(*F* ^2^) = 0.057
*S* = 1.05
3608 reflections
272 parameters
H-atom parameters constrained
Δρ_max_ = 0.56 e Å^−3^
Δρ_min_ = −0.32 e Å^−3^

### 

Data collection: *CrystalClear* (Rigaku/MSC, 2006 ▶); cell refinement: *CrystalClear*; data reduction: *CrystalClear*; program(s) used to solve structure: *SHELXS97* (Sheldrick, 2008 ▶); program(s) used to refine structure: *SHELXL97* (Sheldrick, 2008 ▶); molecular graphics: *SHELXTL* (Sheldrick, 2008 ▶); software used to prepare material for publication: *SHELXTL*.

## Supplementary Material

Crystal structure: contains datablock(s) global, I. DOI: 10.1107/S1600536811032442/pk2337sup1.cif

Structure factors: contains datablock(s) I. DOI: 10.1107/S1600536811032442/pk2337Isup2.hkl

Additional supplementary materials:  crystallographic information; 3D view; checkCIF report

## Figures and Tables

**Table 1:** Hydrogen-bond geometry (Å, °)

*D*—H⋯*A*	*D*—H	H⋯*A*	*D*⋯*A*	*D*—H⋯*A*
O8—H8*W*⋯O9	0.85	1.89	2.732 (3)	170
O6—H4*W*⋯O2′	0.85	2.39	2.905 (19)	119
O5—H1*W*⋯O4^i^	0.85	1.91	2.719 (4)	157
O5—H1*W*⋯O4′^i^	0.85	1.84	2.672 (7)	166
O5—H2*W*⋯O1^ii^	0.85	1.97	2.817 (3)	172
O8—H7*W*⋯O3^ii^	0.85	2.00	2.795 (4)	156
O8—H7*W*⋯O3′^ii^	0.85	2.38	3.127 (15)	147
O6—H3*W*⋯O10^iii^	0.85	1.83	2.680 (3)	178
O6—H4*W*⋯N2^iv^	0.85	2.27	3.025 (3)	148
O7—H5*W*⋯O3^v^	0.85	1.93	2.730 (4)	157
O9—H9*W*⋯O4^v^	0.85	2.00	2.795 (6)	155
O7—H5*W*⋯O3′^v^	0.85	1.91	2.720 (8)	159
O9—H9*W*⋯O3′^v^	0.85	2.06	2.844 (14)	155
O7—H6*W*⋯O9^vi^	0.85	1.97	2.791 (3)	161
O9—H10*W*⋯O1^vii^	0.85	2.06	2.906 (3)	175
O9—H10*W*⋯O4′^vii^	0.85	2.48	3.030 (11)	123
O10—H11*W*⋯N6^viii^	0.85	2.01	2.861 (3)	177
O10—H12*W*⋯O2^ix^	0.85	2.02	2.809 (8)	155
O10—H12*W*⋯O4′^ix^	0.85	2.19	2.944 (14)	148
O10—H12*W*⋯O2′^ix^	0.85	2.51	3.280 (16)	151

Symmetry codes: (i) 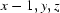; (ii) 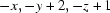; (iii) 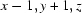; (iv) 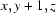; (v) 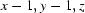; (vi) 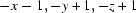; (vii) 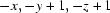; (viii) 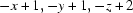; (ix) 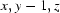.

**Fig. 1.**
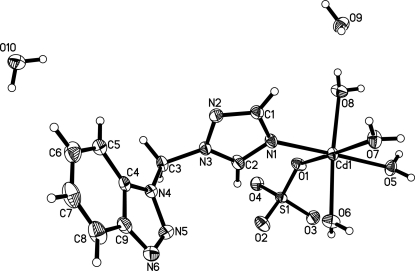
View of the title complex. Displacement ellipsoids are displayed at the 30% probability level. Only one orientation of the disordered SO42- tetrahedron is shown.

**Fig. 2.**
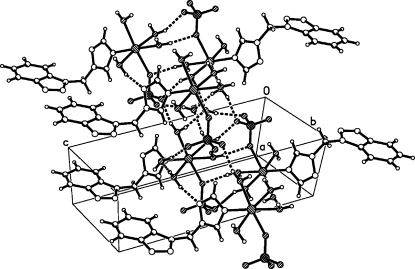
View of hydrogen bonds in the title complex. Hydrogen bonds are indicated by dashed lines.

## supplementary crystallographic information

### Comment

Numerous supramolecular complexes based on triazolyl or benzotriazolyl ligands which have abundant N-donor sites have been synthesized. These show a variety of discrete or infinite frameworks of one-, two-, and three-dimensional motifs (Meng *et al.*, 2009; Yang *et al.*, 2011).  In order to further explore frameworks with new structures, we used 1*H*-1,2,3-benzotriazol-1-yl)methyl]-1*H*-1,2,4-triazole to react with CdSO_4_ at room temperature and obtained the title complex [Cd(SO_4_) (C_9_H_8_N_6_) (H_2_O)_4_] (H_2_O)_2_, which is reported here.  As shown in Fig. 1, the Cd^II^ ion is located in a distorted octahedral coordination environment and is coordinated to five oxygen atoms from four water molecules and one monodentate sulfate anion and one nitrogen atom from the 1*H*-1,2,3-benzotriazol-1-yl)methyl]-1*H*-1,2,4-triazole ligand. Atoms O1, O6, O7, O8 and Cd1 are nearly co-planar (the mean deviation from the plane is 0.0473 Å), O5 and N1 atoms are located in the apical positions. The SO_4_ tetrahedron is rotationally disordered about its S—O axis passing though O1 and S1 atoms. Intramolecular O—H···O hydrogen bonds stabilize the molecular configuration and O—H···O, O—H···N hydrogen bonds between adjacent molecules consolidate the crystal packing (Fig. 2).

### Experimental

The ligand 1*H*-1,2,3-benzotriazol-1-yl)methyl]-1*H*-1,2,4-triazole (0.1 mmol) in methanol (4 ml) was added dropwise to an aqueous solution (3 ml) of cadmium sulfate (0.1 mmol).  The resulting solution was allowed to stand at room temperature. After three weeks colourless crystals of good quality were obtained from the filtrate and dried in air.

### Refinement

The disordered sulfate anion has been modelled by splitting it into two parts (O2, O3, O4 and O2', O3', O4'), the site  occupation factors of which refined in a ratio of 0.651 (12):0.349 (12).  H atoms are positioned geometrically and refined as riding atoms, with C-H = 0.93 Å (aromatic), 0.97 Å (CH_2_) and O-H = 0.85 Å, and with U_iso_(H) = 1.2 U_eq_(C-H) or 1.5 U_eq_(O-H).

### Crystal data

**Table:**

[Cd(SO_4_)(C_9_H_8_N_6_)(H_2_O)_4_]·2H_2_O	*Z* = 2
*M**_r_* = 516.77	*F*(000) = 520
Triclinic, *P*1	*D*_x_ = 1.861 Mg m^−^^3^
Hall symbol: -P 1	Mo *K*α radiation, λ = 0.71073 Å
*a* = 7.7154 (15) Å	Cell parameters from 3156 reflections
*b* = 8.0667 (16) Å	θ = 2.5–27.9°
*c* = 16.369 (3) Å	µ = 1.36 mm^−^^1^
α = 100.12 (3)°	*T* = 293 K
β = 91.64 (3)°	Prism, colourless
γ = 112.38 (3)°	0.19 × 0.17 × 0.14 mm
*V* = 922.3 (3) Å^3^	

### Data collection

**Table:**

Rigaku Saturn CCD diffractometer	3608 independent reflections
Radiation source: fine-focus sealed tube	3361 reflections with *I* > 2σ(*I*)
graphite	*R*_int_ = 0.020
Detector resolution: 28.6 pixels mm^-1^	θ_max_ = 26.0°, θ_min_ = 2.5°
ω scans	*h* = −9→9
Absorption correction: multi-scan (REQAB; Jacobson, 1998)	*k* = −9→9
*T*_min_ = 0.782, *T*_max_ = 0.832	*l* = −19→20
8812 measured reflections	

### Refinement

**Table:**

Refinement on *F*^2^	Primary atom site location: structure-invariant direct methods
Least-squares matrix: full	Secondary atom site location: difference Fourier map
*R*[*F*^2^ > 2σ(*F*^2^)] = 0.025	Hydrogen site location: inferred from neighbouring sites
*wR*(*F*^2^) = 0.057	H-atom parameters constrained
*S* = 1.05	*w* = 1/[σ^2^(*F*_o_^2^) + (0.0278*P*)^2^ + 0.3915*P*] where *P* = (*F*_o_^2^ + 2*F*_c_^2^)/3
3608 reflections	(Δ/σ)_max_ = 0.001
272 parameters	Δρ_max_ = 0.56 e Å^−^^3^
0 restraints	Δρ_min_ = −0.32 e Å^−^^3^

### Special details

**Table:**

Geometry. All e.s.d.'s (except the e.s.d. in the dihedral angle between two l.s. planes) are estimated using the full covariance matrix. The cell e.s.d.'s are taken into account individually in the estimation of e.s.d.'s in distances, angles and torsion angles; correlations between e.s.d.'s in cell parameters are only used when they are defined by crystal symmetry. An approximate (isotropic) treatment of cell e.s.d.'s is used for estimating e.s.d.'s involving l.s. planes.
Refinement. Refinement of *F*^2^ against ALL reflections. The weighted *R*-factor *wR* and goodness of fit *S* are based on *F*^2^, conventional *R*-factors *R* are based on *F*, with *F* set to zero for negative *F*^2^. The threshold expression of *F*^2^ > σ(*F*^2^) is used only for calculating *R*-factors(gt) *etc*. and is not relevant to the choice of reflections for refinement. *R*-factors based on *F*^2^ are statistically about twice as large as those based on *F*, and *R*- factors based on ALL data will be even larger.

### Fractional atomic coordinates and isotropic or equivalent isotropic displacement parameters (Å^2^)

**Table:**

	*x*	*y*	*z*	*U*_iso_*/*U*_eq_	Occ. (<1)
Cd1	−0.08817 (2)	0.82498 (2)	0.629897 (11)	0.02758 (7)	
N1	0.0196 (3)	0.6456 (3)	0.69209 (13)	0.0317 (5)	
N2	0.0601 (3)	0.4169 (3)	0.73914 (14)	0.0350 (5)	
N3	0.2076 (3)	0.5783 (3)	0.76785 (12)	0.0291 (4)	
N4	0.3177 (3)	0.5906 (3)	0.90746 (13)	0.0326 (5)	
N5	0.3149 (4)	0.7462 (3)	0.95407 (15)	0.0458 (6)	
N6	0.2707 (4)	0.7145 (4)	1.02726 (15)	0.0490 (6)	
O1	0.2166 (2)	0.9862 (2)	0.60171 (11)	0.0379 (4)	
O2	0.3964 (14)	1.1563 (16)	0.7348 (7)	0.0486 (17)	0.651 (12)
O3	0.3456 (6)	1.3130 (5)	0.6316 (4)	0.0443 (13)	0.651 (12)
O4	0.5488 (4)	1.1527 (6)	0.6098 (3)	0.0417 (15)	0.651 (12)
O2'	0.345 (2)	1.183 (3)	0.7336 (12)	0.049 (3)	0.349 (12)
O3'	0.438 (2)	1.2932 (10)	0.6056 (5)	0.071 (4)	0.349 (12)
O4'	0.5348 (11)	1.0724 (17)	0.6482 (7)	0.078 (4)	0.349 (12)
O5	−0.1905 (3)	1.0094 (3)	0.56985 (11)	0.0386 (4)	
H1W	−0.2870	1.0265	0.5864	0.058*	
H2W	−0.1868	1.0155	0.5186	0.058*	
O6	−0.0542 (3)	1.0205 (3)	0.75212 (11)	0.0434 (5)	
H3W	−0.1547	1.0216	0.7718	0.065*	
H4W	0.0211	1.1271	0.7482	0.065*	
O7	−0.4009 (2)	0.6650 (3)	0.64632 (13)	0.0440 (5)	
H5W	−0.4523	0.5516	0.6469	0.066*	
H6W	−0.4706	0.7003	0.6187	0.066*	
O8	−0.1160 (3)	0.6613 (3)	0.49667 (11)	0.0438 (5)	
H7W	−0.1616	0.7013	0.4604	0.066*	
H8W	−0.1814	0.5479	0.4928	0.066*	
O9	−0.3463 (3)	0.2982 (3)	0.46744 (13)	0.0448 (5)	
H9W	−0.4038	0.2670	0.5090	0.067*	
H10W	−0.3156	0.2099	0.4472	0.067*	
O10	0.6339 (3)	0.0317 (3)	0.81643 (12)	0.0507 (5)	
H11W	0.6575	0.1056	0.8631	0.076*	
H12W	0.5847	0.0689	0.7802	0.076*	
C1	−0.0485 (4)	0.4643 (3)	0.69349 (16)	0.0334 (6)	
H1A	−0.1625	0.3808	0.6645	0.040*	
C2	0.1805 (4)	0.7115 (3)	0.73996 (16)	0.0351 (6)	
H2A	0.2630	0.8340	0.7523	0.042*	
C3	0.3661 (3)	0.5908 (4)	0.82319 (15)	0.0337 (6)	
H3A	0.4006	0.4879	0.8038	0.040*	
H3B	0.4740	0.7023	0.8218	0.040*	
C4	0.2737 (3)	0.4545 (4)	0.95223 (15)	0.0310 (5)	
C5	0.2629 (4)	0.2757 (4)	0.93464 (18)	0.0397 (6)	
H5A	0.2844	0.2226	0.8828	0.048*	
C6	0.2185 (4)	0.1827 (4)	0.9985 (2)	0.0536 (8)	
H6A	0.2099	0.0626	0.9898	0.064*	
C7	0.1853 (4)	0.2624 (5)	1.0768 (2)	0.0590 (9)	
H7A	0.1538	0.1932	1.1180	0.071*	
C8	0.1983 (4)	0.4385 (6)	1.09375 (19)	0.0569 (9)	
H8A	0.1778	0.4914	1.1458	0.068*	
C9	0.2439 (4)	0.5364 (4)	1.02929 (16)	0.0398 (6)	
S1	0.38047 (8)	1.14858 (8)	0.64711 (4)	0.02752 (13)	

### Atomic displacement parameters (Å^2^)

**Table:**

	*U*^11^	*U*^22^	*U*^33^	*U*^12^	*U*^13^	*U*^23^
Cd1	0.02582 (10)	0.02909 (11)	0.02891 (11)	0.01048 (8)	0.00083 (7)	0.00959 (7)
N1	0.0320 (11)	0.0299 (11)	0.0327 (12)	0.0096 (9)	−0.0026 (9)	0.0116 (9)
N2	0.0355 (12)	0.0249 (11)	0.0421 (13)	0.0093 (9)	−0.0025 (10)	0.0070 (9)
N3	0.0284 (11)	0.0295 (11)	0.0292 (11)	0.0104 (9)	−0.0007 (9)	0.0081 (9)
N4	0.0379 (12)	0.0340 (12)	0.0274 (11)	0.0173 (10)	−0.0031 (9)	0.0037 (9)
N5	0.0579 (15)	0.0407 (14)	0.0426 (14)	0.0280 (12)	−0.0038 (12)	−0.0004 (11)
N6	0.0556 (15)	0.0610 (17)	0.0357 (14)	0.0349 (13)	−0.0019 (11)	−0.0035 (12)
O1	0.0281 (9)	0.0364 (10)	0.0360 (10)	0.0002 (8)	0.0014 (8)	0.0029 (8)
O2	0.052 (4)	0.061 (4)	0.030 (2)	0.019 (3)	−0.002 (3)	0.011 (2)
O3	0.050 (2)	0.0294 (17)	0.054 (3)	0.0158 (16)	−0.0026 (18)	0.0101 (17)
O4	0.0226 (15)	0.046 (2)	0.051 (3)	0.0101 (14)	0.0063 (14)	0.0032 (17)
O2'	0.051 (9)	0.055 (7)	0.027 (4)	0.014 (5)	0.002 (5)	−0.005 (4)
O3'	0.119 (10)	0.027 (4)	0.045 (4)	0.003 (5)	0.019 (5)	0.011 (3)
O4'	0.057 (5)	0.118 (8)	0.097 (7)	0.067 (5)	0.031 (5)	0.044 (7)
O5	0.0441 (11)	0.0479 (12)	0.0365 (10)	0.0281 (9)	0.0057 (8)	0.0174 (9)
O6	0.0446 (11)	0.0376 (11)	0.0372 (11)	0.0065 (9)	0.0068 (9)	0.0023 (8)
O7	0.0288 (10)	0.0359 (11)	0.0651 (13)	0.0060 (8)	0.0016 (9)	0.0205 (10)
O8	0.0536 (12)	0.0360 (11)	0.0327 (10)	0.0095 (9)	−0.0013 (9)	0.0029 (8)
O9	0.0456 (11)	0.0347 (10)	0.0569 (13)	0.0171 (9)	0.0098 (10)	0.0124 (9)
O10	0.0591 (13)	0.0506 (13)	0.0394 (11)	0.0218 (11)	0.0020 (10)	0.0013 (10)
C1	0.0322 (13)	0.0281 (13)	0.0366 (14)	0.0092 (11)	−0.0034 (11)	0.0058 (11)
C2	0.0347 (14)	0.0276 (13)	0.0388 (15)	0.0058 (11)	−0.0059 (11)	0.0123 (11)
C3	0.0294 (13)	0.0456 (16)	0.0292 (13)	0.0164 (12)	−0.0010 (11)	0.0119 (12)
C4	0.0257 (12)	0.0395 (15)	0.0285 (13)	0.0136 (11)	−0.0036 (10)	0.0078 (11)
C5	0.0399 (15)	0.0398 (15)	0.0390 (15)	0.0169 (13)	−0.0044 (12)	0.0056 (12)
C6	0.0485 (18)	0.0426 (18)	0.067 (2)	0.0112 (15)	−0.0058 (16)	0.0220 (16)
C7	0.0457 (18)	0.081 (3)	0.053 (2)	0.0164 (18)	0.0062 (15)	0.0389 (19)
C8	0.0487 (18)	0.099 (3)	0.0331 (16)	0.0346 (19)	0.0138 (14)	0.0239 (17)
C9	0.0332 (14)	0.0583 (19)	0.0291 (14)	0.0214 (14)	0.0001 (11)	0.0048 (13)
S1	0.0238 (3)	0.0256 (3)	0.0280 (3)	0.0054 (2)	0.0010 (2)	0.0029 (2)

### Geometric parameters (Å, °)

**Table:**

Cd1—O6	2.259 (2)	O5—H2W	0.8498
Cd1—O5	2.2733 (18)	O6—H3W	0.8501
Cd1—N1	2.282 (2)	O6—H4W	0.8500
Cd1—O8	2.300 (2)	O7—H5W	0.8500
Cd1—O7	2.3123 (19)	O7—H6W	0.8499
Cd1—O1	2.3190 (19)	O8—H7W	0.8500
N1—C2	1.317 (3)	O8—H8W	0.8500
N1—C1	1.358 (3)	O9—H9W	0.8464
N2—C1	1.309 (3)	O9—H10W	0.8508
N2—N3	1.356 (3)	O10—H11W	0.8499
N3—C2	1.322 (3)	O10—H12W	0.8500
N3—C3	1.462 (3)	C1—H1A	0.9300
N4—N5	1.357 (3)	C2—H2A	0.9300
N4—C4	1.368 (3)	C3—H3A	0.9700
N4—C3	1.440 (3)	C3—H3B	0.9700
N5—N6	1.297 (3)	C4—C9	1.385 (4)
N6—C9	1.378 (4)	C4—C5	1.390 (4)
O1—S1	1.4865 (19)	C5—C6	1.369 (4)
O2—S1	1.425 (11)	C5—H5A	0.9300
O3—S1	1.510 (3)	C6—C7	1.405 (5)
O4—S1	1.442 (3)	C6—H6A	0.9300
O2'—S1	1.45 (2)	C7—C8	1.362 (5)
O3'—S1	1.386 (7)	C7—H7A	0.9300
O4'—S1	1.535 (8)	C8—C9	1.401 (4)
O5—H1W	0.8500	C8—H8A	0.9300
			
O6—Cd1—O5	86.64 (7)	N3—C2—H2A	125.0
O6—Cd1—N1	92.34 (8)	N4—C3—N3	110.7 (2)
O5—Cd1—N1	178.69 (7)	N4—C3—H3A	109.5
O6—Cd1—O8	171.79 (7)	N3—C3—H3A	109.5
O5—Cd1—O8	86.05 (7)	N4—C3—H3B	109.5
N1—Cd1—O8	94.91 (8)	N3—C3—H3B	109.5
O6—Cd1—O7	90.40 (8)	H3A—C3—H3B	108.1
O5—Cd1—O7	86.24 (7)	N4—C4—C9	103.5 (2)
N1—Cd1—O7	94.59 (7)	N4—C4—C5	133.5 (2)
O8—Cd1—O7	92.85 (8)	C9—C4—C5	123.0 (3)
O6—Cd1—O1	92.83 (8)	C6—C5—C4	115.7 (3)
O5—Cd1—O1	90.09 (7)	C6—C5—H5A	122.2
N1—Cd1—O1	89.14 (7)	C4—C5—H5A	122.2
O8—Cd1—O1	83.45 (8)	C5—C6—C7	122.2 (3)
O7—Cd1—O1	174.97 (7)	C5—C6—H6A	118.9
C2—N1—C1	103.1 (2)	C7—C6—H6A	118.9
C2—N1—Cd1	122.73 (17)	C8—C7—C6	121.7 (3)
C1—N1—Cd1	134.18 (17)	C8—C7—H7A	119.2
C1—N2—N3	102.4 (2)	C6—C7—H7A	119.2
C2—N3—N2	110.2 (2)	C7—C8—C9	117.1 (3)
C2—N3—C3	128.2 (2)	C7—C8—H8A	121.5
N2—N3—C3	121.6 (2)	C9—C8—H8A	121.5
N5—N4—C4	111.0 (2)	N6—C9—C4	108.7 (2)
N5—N4—C3	118.8 (2)	N6—C9—C8	130.9 (3)
C4—N4—C3	130.2 (2)	C4—C9—C8	120.4 (3)
N6—N5—N4	107.9 (2)	O3'—S1—O2	128.0 (6)
N5—N6—C9	109.0 (2)	O3'—S1—O4	72.3 (6)
S1—O1—Cd1	135.01 (11)	O2—S1—O4	112.9 (3)
Cd1—O5—H1W	118.7	O3'—S1—O2'	118.7 (9)
Cd1—O5—H2W	124.1	O2—S1—O2'	20.7 (6)
H1W—O5—H2W	108.5	O4—S1—O2'	131.2 (6)
Cd1—O6—H3W	116.9	O3'—S1—O1	113.4 (4)
Cd1—O6—H4W	109.1	O2—S1—O1	112.7 (5)
H3W—O6—H4W	111.7	O4—S1—O1	109.03 (15)
Cd1—O7—H5W	126.0	O2'—S1—O1	108.1 (8)
Cd1—O7—H6W	109.7	O3'—S1—O3	35.5 (6)
H5W—O7—H6W	112.3	O2—S1—O3	108.7 (4)
Cd1—O8—H7W	112.7	O4—S1—O3	107.5 (2)
Cd1—O8—H8W	111.9	O2'—S1—O3	91.6 (7)
H7W—O8—H8W	109.5	O1—S1—O3	105.62 (16)
H9W—O9—H10W	105.1	O3'—S1—O4'	108.7 (6)
H11W—O10—H12W	109.9	O2—S1—O4'	83.7 (4)
N2—C1—N1	114.3 (2)	O4—S1—O4'	37.4 (4)
N2—C1—H1A	122.9	O2'—S1—O4'	104.4 (6)
N1—C1—H1A	122.9	O1—S1—O4'	101.8 (4)
N1—C2—N3	110.0 (2)	O3—S1—O4'	142.0 (5)
N1—C2—H2A	125.0		
			
O6—Cd1—N1—C2	−52.6 (2)	N5—N4—C3—N3	−76.9 (3)
O5—Cd1—N1—C2	−14 (3)	C4—N4—C3—N3	104.2 (3)
O8—Cd1—N1—C2	123.5 (2)	C2—N3—C3—N4	99.9 (3)
O7—Cd1—N1—C2	−143.2 (2)	N2—N3—C3—N4	−78.5 (3)
O1—Cd1—N1—C2	40.2 (2)	N5—N4—C4—C9	−0.1 (3)
O6—Cd1—N1—C1	126.7 (2)	C3—N4—C4—C9	178.9 (2)
O5—Cd1—N1—C1	166 (3)	N5—N4—C4—C5	−177.7 (3)
O8—Cd1—N1—C1	−57.2 (2)	C3—N4—C4—C5	1.2 (5)
O7—Cd1—N1—C1	36.1 (2)	N4—C4—C5—C6	178.0 (3)
O1—Cd1—N1—C1	−140.6 (2)	C9—C4—C5—C6	0.7 (4)
C1—N2—N3—C2	0.9 (3)	C4—C5—C6—C7	0.2 (4)
C1—N2—N3—C3	179.5 (2)	C5—C6—C7—C8	−0.9 (5)
C4—N4—N5—N6	0.0 (3)	C6—C7—C8—C9	0.8 (5)
C3—N4—N5—N6	−179.1 (2)	N5—N6—C9—C4	−0.1 (3)
N4—N5—N6—C9	0.0 (3)	N5—N6—C9—C8	178.7 (3)
O6—Cd1—O1—S1	3.69 (17)	N4—C4—C9—N6	0.1 (3)
O5—Cd1—O1—S1	90.33 (17)	C5—C4—C9—N6	178.1 (2)
N1—Cd1—O1—S1	−88.62 (17)	N4—C4—C9—C8	−178.8 (2)
O8—Cd1—O1—S1	176.35 (17)	C5—C4—C9—C8	−0.8 (4)
O7—Cd1—O1—S1	133.4 (7)	C7—C8—C9—N6	−178.6 (3)
N3—N2—C1—N1	−0.7 (3)	C7—C8—C9—C4	0.1 (4)
C2—N1—C1—N2	0.3 (3)	Cd1—O1—S1—O3'	−118.8 (8)
Cd1—N1—C1—N2	−179.11 (17)	Cd1—O1—S1—O2	36.6 (4)
C1—N1—C2—N3	0.3 (3)	Cd1—O1—S1—O4	162.8 (3)
Cd1—N1—C2—N3	179.79 (15)	Cd1—O1—S1—O2'	15.0 (7)
N2—N3—C2—N1	−0.8 (3)	Cd1—O1—S1—O3	−82.0 (3)
C3—N3—C2—N1	−179.3 (2)	Cd1—O1—S1—O4'	124.6 (6)

### Hydrogen-bond geometry (Å, °)

**Table:**

*D*—H···*A*	*D*—H	H···*A*	*D*···*A*	*D*—H···*A*
O8—H8W···O9	0.85	1.89	2.732 (3)	170.
O6—H4W···O2'	0.85	2.39	2.905 (19)	119.
O5—H1W···O4^i^	0.85	1.91	2.719 (4)	157.
O5—H1W···O4'^i^	0.85	1.84	2.672 (7)	166.
O5—H2W···O1^ii^	0.85	1.97	2.817 (3)	172.
O5—H2W···S1^ii^	0.85	2.89	3.616 (2)	145.
O8—H7W···O3^ii^	0.85	2.00	2.795 (4)	156.
O8—H7W···O3'^ii^	0.85	2.38	3.127 (15)	147.
O6—H3W···O10^iii^	0.85	1.83	2.680 (3)	178.
O6—H4W···N2^iv^	0.85	2.27	3.025 (3)	148.
O7—H5W···O3^v^	0.85	1.93	2.730 (4)	157.
O7—H5W···S1^v^	0.85	3.01	3.856 (2)	178.
O9—H9W···O4^v^	0.85	2.00	2.795 (6)	155.
O9—H9W···S1^v^	0.85	2.92	3.770 (2)	177.
O7—H5W···O3'^v^	0.85	1.91	2.720 (8)	159.
O9—H9W···O3'^v^	0.85	2.06	2.844 (14)	155.
O7—H6W···O9^vi^	0.85	1.97	2.791 (3)	161.
O9—H10W···O1^vii^	0.85	2.06	2.906 (3)	175.
O9—H10W···S1^vii^	0.85	2.88	3.667 (2)	155.
O9—H10W···O4'^vii^	0.85	2.48	3.030 (11)	123.
O10—H11W···N6^viii^	0.85	2.01	2.861 (3)	177.
O10—H12W···O2^ix^	0.85	2.02	2.809 (8)	155.
O10—H12W···S1^ix^	0.85	2.95	3.796 (2)	173.
O10—H12W···O4'^ix^	0.85	2.19	2.944 (14)	148.
O10—H12W···O2'^ix^	0.85	2.51	3.280 (16)	151.

Symmetry codes: (i) *x*−1, *y*, *z*; (ii) −*x*, −*y*+2, −*z*+1; (iii) *x*−1, *y*+1, *z*; (iv) *x*, *y*+1, *z*; (v) *x*−1, *y*−1, *z*; (vi) −*x*−1, −*y*+1, −*z*+1; (vii) −*x*, −*y*+1, −*z*+1; (viii) −*x*+1, −*y*+1, −*z*+2; (ix) *x*, *y*−1, *z*.
